# Opposing roles of PlexinA and PlexinB in axonal branch and varicosity formation

**DOI:** 10.1186/1756-6606-4-15

**Published:** 2011-04-13

**Authors:** Shay Q Neufeld, Alexa D Hibbert, Brian E Chen

**Affiliations:** 1 Research Institute of the McGill University Health Centre, Centre for Research in Neuroscience, Montréal, Québec, Canada; 2 Departments of Medicine and Neurology & Neurosurgery, McGill University, Montréal, Québec, Canada

## Abstract

Establishing precise synaptic connectivity during development is crucial for neural circuit function. However, very few molecules have been identified that are involved in determining where and how many synapses form. The Plexin cell-surface molecules are a conserved family of axon guidance receptors that mediate axon fasciculation and repulsion during neural development, and later in development PlexinA receptors are involved in eliminating axonal branches and synapse numbers. Here we investigate the roles of PlexinA and PlexinB receptors in axonal branch and varicosity formation in *Drosophila*. We knocked down PlexinA or PlexinB expression using RNAi in identified mechanosensory neurons and analyzed axonal branching patterns and varicosity formations. Reducing PlexinA expression increased the axonal arbor complexity by increasing the number of branches and varicosities along the axon. In contrast, knocking down PlexinB expression decreased morphological complexity by decreasing the number of branches and the overall size of the axonal arbor, but did not reduce the number of varicosities. Our results demonstrate opposing roles for PlexinA and PlexinB in local wiring within a target region, where PlexinA functions to suppress excessive axonal branches and synapses and PlexinB facilitates axonal growth.

## Background

The intricate connectivity of neural circuits is formed during development by growing axons that must integrate a complex array of growth cues and guidance signals to innervate specific targets. Several receptor families and guidance cues involved in axon navigation to target regions have been uncovered, but the molecular mechanisms underlying local targeting decisions remain unclear. Many of the molecules involved in axon guidance to target regions have also been found to function in regulating synaptic connectivity and synapse formation [1-4]. For example, a knockout mouse of the axon guidance receptor PlexinA4 exhibits a variety of defects in its embryonic peripheral and central nervous systems, such as abnormal trajectories of cranial and spinal nerves, and overshooting of ophthalmic nerve fibers past their peripheral target fields [5]. At later post-natal ages, the PlexinA3 and PlexinA4 receptors were found to also mediate axonal pruning of branches and collaterals and elimination of synaptic complexes [6,7].

The Plexins are a family of neuronal receptors conserved across vertebrates and invertebrates. Vertebrates have nine Plexins grouped into four classes (A-D) based on homology [8]. *Drosophila *have two Plexins belonging to class A and class B, encoded by the *PlexA *and *PlexB *genes [9]. *Drosophila *PlexinA and PlexinB have shared and distinct roles in mediating axon guidance during embryogenesis. Both PlexinA and PlexinB null mutants have similar guidance defects where motor axons fail to defasciculate properly to innervate wing muscle [10]. However, different longitudinal nerve bundles require different Plexins to fasciculate properly within the central nervous system (CNS) [10]. PlexinA and PlexinB also bind distinct ligands in flies: PlexinA is the receptor for the class 1 transmembrane Semaphorin ligands, Sema1a and Sema1b, whereas PlexinB binds the class 2 secreted Sema2a ligand [9-11]. Plexins direct axons by activation of cytoskeletal reorganization through the Rho family of small GTPases, and the active form of the GTPase Rac1 has been shown to directly bind the cytoplasmic tails of both PlexinA and PlexinB [12-15].

Although the class A Plexins in mammals have been implicated in axonal branch pruning, it is not known whether *Drosophila *PlexinA might be involved in a similar manner in branch and synapse formation, and what role, if any, PlexinB has beyond axon guidance. Here we compare the roles of *Drosophila *PlexinA and PlexinB in the axon guidance of peripheral mechanosensory axons for their choice of a specific pathway into the CNS. We also examine the roles of PlexinA and PlexinB in axonal branching within a CNS target region. We imaged single mechanosensory axons projecting into the thoracic ganglion, and analyzed their axonal morphologies after RNAi knockdown of *PlexA *or *PlexB*. We show that PlexinA, but not PlexinB is required for proper axon guidance into the CNS. Using quantitative analysis of the axonal arbor, we also show that decreasing PlexinA expression results in increased axonal arbor complexity and axonal contacts, whereas decreasing PlexinB expression decreases arbor complexity. Our data suggest a model where PlexinA suppresses branch formation, and PlexinB promotes axonal branching during circuit formation.

## Results

**Figure 1:**
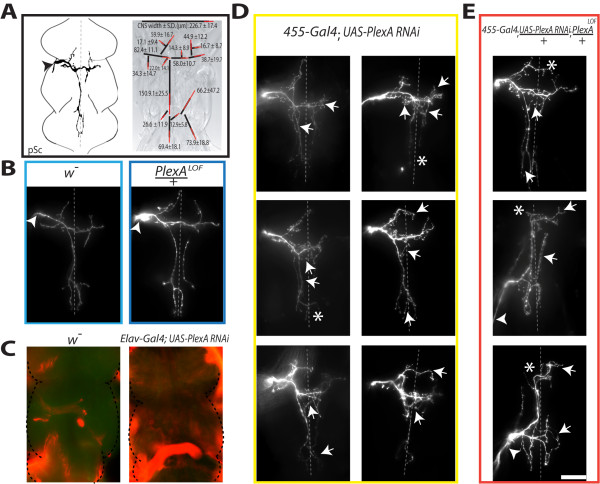
**Reducing PlexinA expression increases axonal branching**. A, The posterior scutellar (pSc) neuron has a stereotyped axonal branching pattern within the central nervous system. Left panel, the pSc mechanosensory neuron enters the thoracic ganglion (arrowhead) in a specific nerve bundle within the mesothoracic region. Right panel, the pSc axonal arbor consists of 16 stereotyped, "skeleton" branches (black lines), and 6 additional variable branches (not shown). Length measurements are shown in micrometers with standard deviations of individual branches indicated as red lines. B, Axon guidance into the thoracic ganglion is unaffected in *PlexA*^*LOF*^/+ heterozygous mutants. C, PlexinA is broadly distributed throughout the thoracic ganglion. PlexinA protein expression is shown in green with MAP1B protein counterstained in red. Pan-neuronal knockdown of PlexinA using RNAi reduces PlexinA protein to undetectable levels. D, RNAi-mediated knockdown of PlexinA in the pSc axon increases the number of axonal branches (arrows). Selective expression of dsRNA constructs within pSc neurons was through the *455-Gal4 *driver. E, Combining PlexinA RNAi in pSc neurons with *PlexA*^*LOF *^mutants increases axon guidance errors (arrowheads), routing errors (asterisks), and excessive branching (arrows). Dotted lines represent the midline and the scale bar is 50 μm.

To examine the role of the Plexin molecules in axon guidance and synaptic targeting, we used the *Drosophila *mechanosensory system. This system is a good model for studying targeting because of the invariant axonal targeting patterns of individually identifiable neurons [16-18]. We focused on the left and right pair of posterior mechanosensory neurons on the fly scutellum, called the posterior scutellar (pSc) neurons. The pSc axon enters the thoracic ganglion via the posterior dorsal mesothoracic nerve bundle (arrowhead in Figure 1A) and elaborates a stereotyped branching pattern to synapse with interneurons within the CNS [17]. We imaged the axon entry point and axonal branching patterns of single pSc neurons by labeling them with fluorescent dyes (Figure 1). In wildtype flies, the pSc axon guidance into the CNS is remarkably precise and we found no instance of axon guidance errors. To determine the wildtype variability in targeting of branches within the CNS, we measured the number and lengths of branches in 21 wildtype flies, and identified a "core" pattern of 16 specific branches that were present in more than 80% of animals (Figure 1A). These 16 pSc axonal branches were designated as the prototypical pSc skeleton. Thus, the average pSc neuron has 16 skeletal branches and 6 additional variable branches that make up its total axonal arbor.

Loss of function of either of the two *Drosophila *Plexin genes, *PlexA *and *PlexB*, is homozygous lethal in adults [9-11,19]. Therefore, we first characterized heterozygous animals of a *PlexA*^*f05547 *^loss of function (LOF) allele, *PlexA*^*LOF*^/+. We examined the pSc axonal pathway choice in the *PlexA*^*LOF*^/+ mutants to assess its effects on axon guidance into the CNS (Figure 1B). We found that in all *PlexA*^*LOF*^/+ heterozygous mutants, the pSc axon entered the thoracic ganglion at the proper position in the mesothoracic segment (arrowheads in Figure 1B) and had no occurrences of axon guidance errors, similar to wildtype. Interestingly, in analyzing the axonal arbors within the CNS, we observed a significant increase in the number of pSc axonal branches (26.5 ± 0.7 branches, *n *= 15) in *PlexA*^*LOF*^/+ heterozygous mutants compared to *w*^- ^wildtype controls (22.5 ± 0.3 branches, *n *= 21) (*p *< 0.05).

We examined the expression pattern of PlexinA within the thoracic ganglion, using antibodies against PlexinA protein and the MAP1B cytoskeletal protein as a neuronal marker (Figure 1C). PlexinA protein was observed in a punctuate, dashed pattern presumably along neuronal processes (Additional file 1, Figure S1A) and overlapped with MAP1B, widely expressed throughout the ganglion. We confirmed this expression to be pre-dominantly neuronal, as a pan-neuronal (*elav-Gal4*) knockdown of PlexinA using RNAi reduced PlexinA protein levels to near undetectable levels (Figure 1C and Additional file 1, Figure S1B). A previous report of PlexinA expression during development has shown it to be similar and broadly distributed throughout the CNS in motor and sensory axons [9]. Thus, to further characterize how PlexinA is involved in pSc axonal targeting, we moved to a mosaic analysis of PlexinA function.

### PlexinA is Required for Precise Axonal Arbor Formation and Suppresses Branch Growth

We wanted to know whether the increase in the number of axonal branches in the PlexinA heterozygous mutants was through PlexinA's cell-autonomous functions within the pSc neuron. However, mosaic analysis of PlexinA or PlexinB function in *Drosophila *has proven difficult because both genes are located on the small fourth chromosome where recombination does not occur, precluding standard genetic techniques to manipulate Plexins [19]. We therefore turned to the Gal4/UAS system to express double-stranded "hairpin" RNA to trigger RNAi [20-23]. We confirmed the efficacy of *PlexA *RNAi knockdown using semi-quantitative analysis of immunohistochemistry against PlexinA compared to MAP1B levels (Additional file 1, Figure S1B). We induced RNAi knockdown of *PlexA *early in development of pSc neurons using a scutellar Gal4 driver, *455-Gal4*, to activate the expression of UAS-dsRNA constructs [24].

We found that 14% of pSc axons in *455-Gal4*; *UAS-PlexA-dsRNA *flies (*n *= 37) entered the thoracic ganglion at the incorrect position. However, this frequency of axon guidance errors was not significantly different from those found in control *455-Gal4 *flies (Table 1 and **Methods**). Within the CNS, however, pSc axons with PlexinA RNAi knockdown produced extensive supernumerary branches, and had approximately 7 more branches as compared to *455-Gal4 *controls (31 ± 0.8 versus 24.4 ± 0.7, respectively, *p *< 0.001) (Figure 1D). The growth of these extra branches significantly increased the total axonal arbor size in PlexinA RNAi flies by 17% to 1219 ± 32 μm (S.E.M.) compared to 1045 ± 31 μm in controls (*p *< 0.01). We also observed frequent branch routing errors of the identifiable pSc skeletal branches (asterisks in Figure 1D) indicating a loss of targeting within the pSc axonal branches. This mosaic analysis demonstrates that PlexinA is required solely within the pSc axon to prevent excessive branching and targeting errors within the CNS.

**Table 1:** Quantitation of the morphological analyses performed on each genotype.

Genotype	N	Frequency of Axon Guidance Errors	# of Branches	Total Arbor Size	# of Varicosities
*w-*	21	0%	22.5 ± 0.3	906 ± 19 μm	28.3 ± 4

*455-Gal4*	20	15%	24.4 ± 0.7	1045 ± 31 μm	35.7 ± 7

*PlexA*^*LOF*^/+	15	6%	26.5 ± 0.7	990 ± 131 μm	35.5 ± 5

*455-Gal4*; *UAS-PlexA-dsRNA*	37	14%	31 ± 0.8	1219 ± 32 μm	68.2 ± 13

*455-Gal4*; *UAS-PlexA-dsRNA*/+; *PlexA*^*LOF*^/+	10	56% (n = 18)	31 ± 2.0	1305 ± 49 μm	79.8 ± 14

*PlexB*^*LOF*^/+	21	19%	22.7 ± 0.6	902 ± 23 μm	36.1 ± 13

*455-Gal4*; *UAS-PlexB-dsRNA*	42	5%	21.5 ± 0.7	918 ± 28 μm	37.4 ± 8

*455-Gal4*; *UAS-PlexB-dsRNA*/+; *PlexB*^*LOF*^/+	20	15%	21.9 ± 0.9	897 ± 30 μm	34 ± 10

*455-Gal4*; *UAS-PlexA-dsRNA*/*UAS-PlexB-dsRNA*	16	0%	30.9 ± 2	1161 ± 54 μm	40.9 ± 10

**Figure 2:**
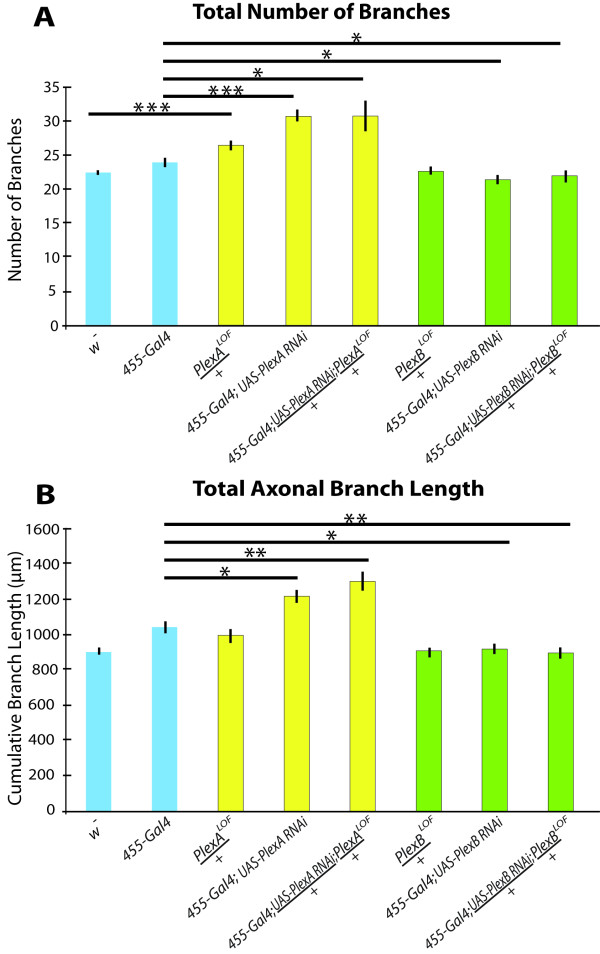
**Reducing PlexinA in axons produces opposite effects from reducing PlexinB**. A, The pSc axonal arbors in PlexinA mutants have significantly more branches compared to controls. RNAi of PlexinB in pSc neurons using the *455-Gal4 *driver significantly reduced the number of branches compared to *455-Gal4 *controls. B, PlexinA RNAi in pSc neurons increased the total axonal arbor size, and PlexinB RNAi in pSc neurons decreased the arbor size. Combining the RNAi in pSc neurons with the heterozygous mutant enhanced the RNAi phenotype compared to *455-Gal4 *controls, but was not significantly different from RNAi in pSc neurons alone. 1 asterisk indicates statistically significant differences with *p *values less than 0.05, 2 asterisks represent *p *values less than 0.01, and 3 asterisks represent *p *values less than 0.001. Measurements in all PlexinA mutants are significantly greater than measurements in all PlexinB mutants in both graphs, and are not indicated for clarity.

When we combined PlexinA RNAi and *PlexA*^*LOF*^/+ flies, we found that all *455-Gal4*; *UAS-PlexA-dsRNA*/+; *PlexA*^*LOF*^/+ flies had routing errors and excessive branches in the pSc axon (Figure 1E). The enhancement of the excessive branching and misrouting phenotypes was so severe that only 10 out of 18 flies were analyzable due to the number of overlapping and intertwined branches. The number of pSc axonal branches and total arbor size (31 ± 2.0 and 1305 ± 49 μm, respectively) in these flies were significantly greater than control, but were not significantly different from PlexinA RNAi within the pSc alone (Figure 2). The *455-Gal4*; *UAS-PlexA-dsRNA*/+; *PlexA*^*LOF*^/+ flies did have a substantial increase in axon guidance errors with 56% entering the CNS incorrectly. The pSc axon guidance errors entered into the haltere nerve pathway at a posterior entry point in the metathoracic neuromere (arrowheads, Figure 1E) instead of into the mesothoracic neuromere. These results indicate that the finer scale branching decisions are more sensitive to PlexinA expression than axon guidance decisions, because we observed an increase in branch number even in heterozygous PlexinA mutants. As PlexinA levels are progressively reduced, axonal arbor complexity increases, demonstrating its critical role in suppressing or destabilizing inappropriate branches.

### PlexinB Promotes Axonal Growth

**Figure 3:**
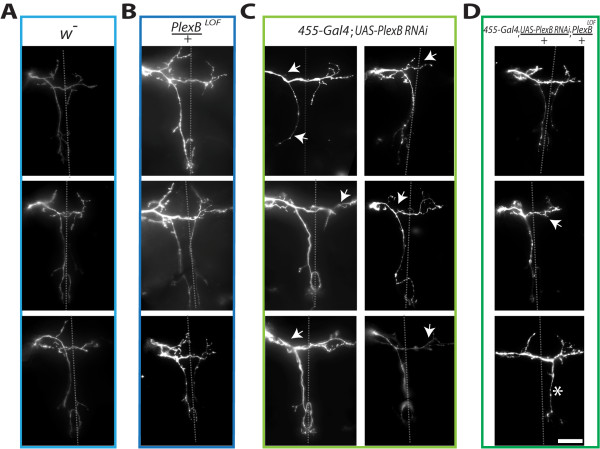
**PlexinB knockdown decreases axonal complexity**. A, Wildtype flies have a stereotyped pSc axonal branching pattern. B, *PlexB*^*LOF*^/+ do not have axon guidance errors and have wildtype branching patterns. C, Reduced PlexinB expression in pSc neurons results in fewer branches, and an overall decrease in arbor growth. Arrows denote missing stereotypical skeleton branches of the pSc arbor that normally occur in wildtype flies. D, Combining PlexinB RNAi with *PlexB*^*LOF*^/+ phenocopies the *455-Gal4; UAS-PlexB-RNAi *pSc axons, and does not induce axon guidance errors. Dotted lines represent the midline and the scale bar is 50 μm.

PlexinB has been shown to share similar functions as PlexinA during embryonic development in axon defasciculation in motor nerves and in axon fasciculation in CNS nerve bundles [9,10,25]. However, PlexinB is required in a specific medial nerve bundle for fasciculation in the embryonic CNS, whereas PlexinA is required in lateral bundles [9,10]. To determine whether PlexinB might also have distinct functions from PlexinA in mechanosensory axonal arbor formation, we analyzed pSc arbors in *455-Gal4*; *UAS-PlexB-dsRNA *flies (Figure 3). Interestingly, we found that *PlexB *RNAi reduced the pSc axonal arbor complexity by decreasing the total number of branches and total arbor size, both by 12%. The number of branches was reduced from 24.4 ± 0.7 in controls to 21.5 ± 0.7 in PlexinB RNAi pSc neurons (*p *< 0.05). This loss of branches decreased the total axonal arbor size from 1045 ± 31 μm in controls to 918 ± 28 μm with PlexinB knockdown (*n *= 42) (*p *< 0.01) (Figure 2). This significant decrease in axonal arbor size was maintained when PlexinB RNAi was crossed to *PlexB*^*KG00878 *^LOF animals (*455-Gal4*; *UAS-PlexB-dsRNA*/+; *PlexB*^*LOF*^/+, 897 ± 30 μm, *n *= 20, *p *< 0.01), and was not different from PlexinB RNAi alone (Figure 3D and Figure 2). However, in comparing *PlexB*^*LOF*^/+ flies to *w*^- ^controls, we found no significant differences in pSc axonal branch number or in axonal arbor sizes. PlexinB was not required for axon guidance into the CNS, as none of the PlexinB mutants had significantly different rates of guidance errors compared to control. Thus, in contrast to PlexinA, loss of PlexinB decreases the number of axonal branches and reduces axonal arbor size, and does not affect pSc axon pathway choice (Figure 2 and Table 1). Because the combination of *PlexB*^*LOF*^/+ heterozygous flies with PlexinB RNAi knockdown in the pSc phenocopied the PlexinB RNAi knockdown in the pSc alone in terms of both decreased branching and branch growth, these results indicate an efficient targeting of the *PlexB *RNA using RNAi and efficient knockdown of PlexinB.

**Figure 4:**
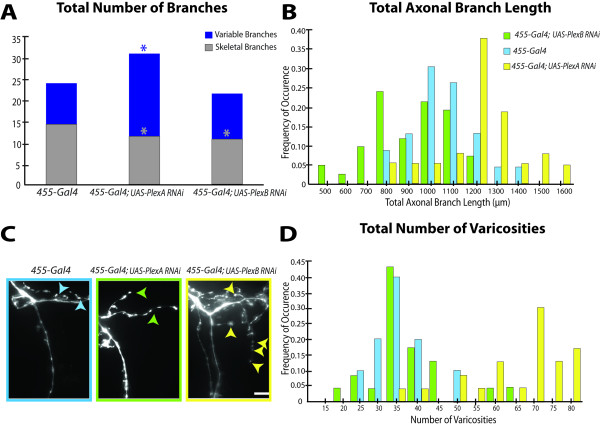
**Quantitative analysis of PlexinA and PlexinB branches and varicosities reveals opposing roles in local axonal growth and synapse formation**. A, PlexinA RNAi within the pSc neuron increases the number of variable axonal branches, whereas PlexinB RNAi decreases the number of highly stereotyped (skeletal) branches. Significant decreases in the number of highly skeletal pSc branches were also observed in the PlexinA RNAi neurons due to an increase in branch routing errors. Asterisks denote significance of *p *< 0.05. B, The pSc axonal arbor sizes with PlexinA RNAi are significantly shifted towards larger values. The distribution of pSc arbor sizes with PlexinB RNAi on the other had is shifted towards smaller values. Frequency distributions of the total axonal branch lengths among the three genotypes, *455-Gal4*, and *455-Gal4*; *UAS-PlexA-RNAi*, and *455-Gal4; UAS-PlexB-RNAi *were significantly different from each other (*p *< 0.05). The average arbor size for *455-Gal4 *control flies was 1045 μm and approximately 30% of flies had total branch lengths of 1000 μm (blue bars). The average arbor size in PlexinB RNAi pSc neurons was 918 μm with 25% of the neurons having total branch lengths of 800 μm (green bars). Reducing PlexinA expression in pSc neurons had the opposite effect, and increased their average total branch lengths to 1219 μm with 35% of arbors at 1200 μm (yellow bars). Histogram bin width, 100 μm. C, Quantification of axonal varicosities is an indirect measure of synaptic contacts. Varicosities (arrowheads) along branches were counted to obtain a lower estimate of synapse number per pSc axon. Scale bar, 5 μm. D, PlexinA knockdown significantly increases varicosities along the pSc axon. Frequency distribution of the number of varicosities in *455-Gal4*; *UAS-PlexA-RNAi *pSc axons (yellow bars) was shifted to the right compared to the *455-Gal4 *distribution (blue bars) (*p *< 0.01), with no significant difference in *455-Gal4*; *UAS-PlexB-RNAi *(green bars) compared to control.

To examine how reducing PlexinB expression decreased the axonal arbor, we analyzed individually identifiable branches of the pSc axonal arbor. Axonal branches can be classified as being either a skeletal branch that is one of the core branches of the pSc, or a variable branch that is not part of the pSc skeleton. Analysis of the PlexinB RNAi pSc neurons revealed a significant loss of an average 3.3 branches missing from the pSc skeleton (Figure 4A) (*p *< 0.01). A decrease in skeletal branches was also observed in PlexinA RNAi pSc neurons, but this was due to the frequent branch misrouting phenotypes observed in PlexinA mutants where skeletal branches could no longer be identified with certainty (asterisks in Figure 1), and was accompanied by a large increase in variable branches (8.6 more variable branches than *455-Gal4*, *p *< 0.001) (Figure 4A). PlexinB RNAi axons, however, did not have a significant increase in the number of variable branches. When we measured the total lengths of the skeletal branches in PlexinB RNAi pSc neurons, we also found a significant decrease in size (*p *< 0.001). The combined decreases in number of axonal branches and size of branches shifted the distribution of total axonal lengths in PlexinB RNAi neurons to smaller values (*p *< 0.05) (Figure 4B). Conversely, excessive branch formation in PlexinA RNAi neurons significantly shifted the total axonal length distribution towards much larger values than controls (*p *< 0.01) (Figure 4B).

### PlexinA Suppresses Axonal Varicosity Formation

We wanted to determine whether the changes in axonal arbor growth in the PlexinA and PlexinB mutants affected synapse formation within the pSc axon. Therefore, we quantified the number of axonal varicosities along the pSc axon as an estimate of synapse number and location in a single neuron [26,27] (Figure 4C). To ensure that the varicosities counted were likely to be synaptic boutons, we focused on the largest varicosities along the axon to estimate a lower bound on the number of pre-synaptic sites (see **Methods**). PlexinA RNAi pSc axons were found to have almost double the number of varicosities with 68.2 ± 13 compared to 35.7 ± 7 in *455-Gal4 *(*p *< 0.001) (Figure 4D). Excessive varicosities were found throughout the ectopic branches, potentially making inappropriate synaptic contacts. Quantification of varicosities in PlexinB RNAi axons however, showed no significant difference compared to controls (37.4 ± 8, *p *> 0.05) (Figure 4D). To confirm that the changes in varicosity numbers and branches were due to a loss of PlexinA specifically in the pSc neurons, we co-labeled another neighboring mechanosensory neuron, the posterior Dorsocentral (pDc) (Additional file 2, Figure S2A). In *455-Gal4*; *UAS-PlexA-dsRNA *flies, we found that as expected, the pSc neurons had extensive branches and varicosities, but the pDc neuron displayed a wildtype arbor even though both axons encountered similar targets within the CNS (Additional file 2, Figure S2A).

### PlexinA and PlexinB Have Opposing Roles in Axon Growth

**Figure 5:**
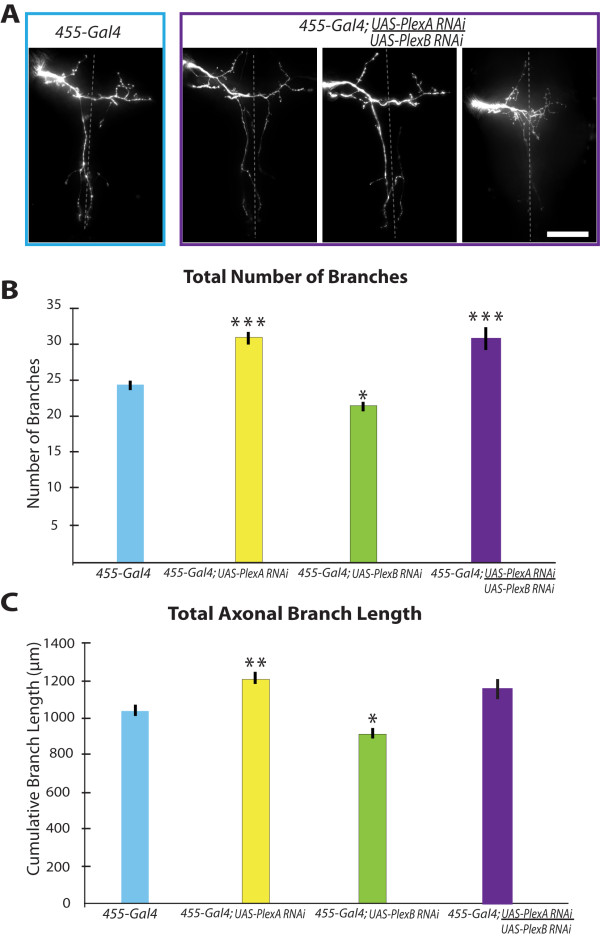
**Simultaneous reduction of PlexinA and PlexinB in a single pSc neuron produces both phenotypes**. A, Simultaneous expression of *PlexA-dsRNA *and *PlexB-dsRNA *within a single pSc neuron results in both wildtype branching patterns and PlexinA mutant branching patterns. Scale bar, 50 μm. B, C, Double knockdown of PlexinA and PlexinB within pSc neurons significantly increases the number of axonal branches, but does not significantly affect the total arbor size.

If PlexinA and PlexinB have opposing roles in axonal branch elaboration, which mechanism dominates if both are knocked down in a single neuron? We co-expressed PlexinA RNAi and PlexinB RNAi in pSc neurons driven by *455-Gal4 *(i.e., *455-Gal4*; *UAS-PlexA-dsRNA*/*UAS-PlexB-dsRNA*). We found that these neurons were not significantly different from control flies in branch lengths and varicosities, but there was a significant increase in branch numbers (*p *< 0.001) (Figure 5). These results may be due to a partial penetrance of both phenotypes where the loss of PlexinA increased the number of branches, but the loss of PlexinB significantly reduced the skeletal branch lengths (*p *< 0.05) and possibly the lengths of the variable branches. This supports the hypothesis that the two Plexins function distinctly and in opposition from each other.

## Discussion

Previous studies have indicated redundant roles for the two Plexin molecules in axon guidance, as well as distinct but highly similar roles in guidance and fasciculation [9,10]. In our study, we used RNAi in single mechanosensory neurons in combination with genetic and quantitative morphological analyses to demonstrate opposing functions for PlexinA and PlexinB in local axonal branch and varicosity formation. We found that decreased PlexinA expression within mechanosensory axons caused excessive branching and varicosities. Decreasing PlexinB expression reduced the number of branches and size of the axonal arbor. Our results demonstrate a novel role for *Drosophila *Plexins that is independent of axon guidance functions. We did observe significant axon guidance errors in 56% of *455-Gal4*; *UAS-PlexA-dsRNA*/+; *PlexA*^*LOF*^/+ flies, whereas in PlexinB mutants pathfinding errors did not significantly occur. These results are consistent with a previous report that axon guidance defects in PlexinA mutants cannot be compensated for by PlexinB, but guidance defects in PlexinB mutants can be partially rescued by PlexinA [10], however we did not observe any axon guidance errors in double mutant PlexinA and PlexinB pSc neurons (Figure 5 and Table 1). Pathway choice for axonal entry into the CNS is a highly stereotyped process, but the mechanisms involved in axonal fasciculation into and defasciculation out of nerve bundles have thus far only been characterized in homeotic mutations, and physical manipulations [17,28,29]. Our results demonstrate PlexinA as a guidance molecule required for specifying pathway choice of mechanosensory axons into the CNS.

Our results also uncovered a role for PlexinA in axonal arbor formation within the CNS by suppressing or destabilizing axonal branches and varicosities. These results are consistent with previous experiments that have shown that mammalian PlexinA3 and PlexinA4 are involved in axonal branch pruning and synapse elimination [6,7]. *Drosophila *PlexinA does not seem to operate in an identical manner, however, in that the stereotyped pSc axonal arbor in PlexinA mutants is not formed with an excess of branches that normally would have been pruned, as is the case for mammalian PlexinA. Instead, PlexinA mutant axons have frequent branch routing errors in addition to excessive branching, indicating that PlexinA is not simply involved in branch pruning but may also be involved in targeting (Figure 1). We also observed an increase in axonal varicosities in the branches of PlexinA mutant neurons which suggests two possibilities: Either that the formation of a new branch necessitates synapse formation along the branch, regardless of PlexinA, or that PlexinA is required for the active suppression of synapses as well as branches. We favor the latter hypothesis based on analysis of varicosity numbers per branch in PlexinA mutants that revealed a 54% increase in the number of varicosities per branch (Additional file 2, Figure S2B). This suggests that PlexinA may be involved in suppressing synapse formation as well as branch formation in axons.

Plexin receptors are associated with axon repulsion and growth cone collapse. How does *Drosophila *PlexinB in mechanosensory axons induce branch growth within the CNS? Our findings that PlexinB knockdown in pSc neurons decreased axonal growth are consistent with previous experiments *in vitro *showing that human PlexinB2 and PlexinB3 could promote neurite outgrowth [30]. These experiments also demonstrated that the class B Plexins could bind homophilically *in trans *across membranes, providing a potential Semaphorin-independent mechanism by which Plexins could promote growth.

How might the two similar cell-surface receptors PlexinA and PlexinB mediate opposing effects on axonal branch growth? Two different mechanisms may exist to allow for opposing effects of PlexinA and PlexinB: differential binding of the Plexins to specific Semaphorin ligands, or Semaphorin-dependent activation of PlexinA and Semaphorin-independent activation of PlexinB through homophilic interactions *in trans*. Evidence for differential binding exists from the crystal structures of vertebrate Plexin-Semaphorin complexes [31,32]. These results show that PlexinA2 homodimerizes *in cis *to bind to homodimers of Sema6A, while PlexinB1 homodimerizes to bind SEMA4D homodimers, with their binding specificity conferred via conserved interactions between the sema domains in both Plexin and Semaphorin [31,32]. Furthermore, experiments examining *Drosophila *PlexinA and PlexinB function have shown that the differential binding affinities of PlexinA for the Sema1a ligand and PlexinB binding Sema2a controls precise synaptic patterning through expression of Plexins and Semaphorins in specific neuronal subsets [9-11]. Specific targeting of sensory axons was found to be dependent on different gradients of Semaphorins.

However, the specificity for PlexinA and PlexinB to bind the class 1 and class 2 Semaphorins, respectively, is not clear [for example see Figure S9 in reference 11], and our genetic interaction experiments examining class 2 Semaphorins and PlexinB within the mechanosensory system have not demonstrated any significantly different phenotypes (unpublished observations). In addition, PlexinB has been also shown to bind PlexinA and some of its axon guidance functions occur through the same signaling molecules (e.g., MICAL) as PlexinA [10]. Intracellular signaling molecules downstream of surface receptors have not been fully identified, and how they interact to control branch growth is still poorly understood [33-38]. Thus, PlexinA expressed in mechanosensory axons may detect Sema1a or Sema1b expressed on inappropriate targets resulting in branch retraction, and secreted Sema2a in the surrounding environment may be detected by PlexinB in axonal branches resulting in growth. Alternatively, PlexinA with or without PlexinB in a complex may detect Semaphorins to induce branch retraction, but if PlexinB is detected *in trans*, axonal growth occurs. It will be interesting to see whether mechanosensory axons utilize Plexin receptors through ligand-specific interactions or through homophilic interactions, and the downstream signaling mechanisms underlying branch and synapse growth and suppression.

## Conclusions

We used the stereotyped axonal arbors of the pSc mechanosensory neuron to examine the roles of PlexinA and PlexinB receptors in axonal branching and varicosity formation. Our study shows that PlexinA is required for suppression of excess axonal branches and varicosities, and that PlexinB is involved in branch growth. These findings demonstrate that the PlexinB receptor is not redundant for PlexinA function, but acts in opposition. These results will aid our understanding of the molecular cues involved in determining how many and where synapses should form during neural circuit development and provide molecular targets for promoting axonal branch growth and synapse formation.

## Methods

### *Drosophila *Genotypes

*PlexA*^*f05547 *^and *PlexB*^*KG00878 *^mutants were obtained from the Bloomington Fly Center, and were characterized as loss of function alleles, or strong hypomorphs by their failure to complement the deficiency lines *Df(4)C3 *and *Df(4)M101-62f*, respectively, for rescue of adult lethality [10,39]. The GAL4-UAS system was used to express dsRNA to induce RNA interference (RNAi) in specific cells [23]. The pan-neuronal driver *elav-Gal4 *and the eye-specific *GMR-Gal4 *were obtained from the lab of Dr. Yong Rao. We used the Gal4 line *455-Gal4 *to selectively express dsRNA in the scutellum, which includes four (right, left, anterior, and posterior) scutellar mechanosensory neurons [24]. UAS-dsRNA for *PlexA *and *PlexB *were obtained from the Vienna Drosophila RNAi Centre (VDRC) and the lab of Dr. Yong Rao [19,22]. From the VDRC stock center, one UAS-PlexA RNAi line (transformant ID 4740) and six UAS-PlexB RNAi lines (transformant IDs 46687, 12167, 8383, 21279, 27220, 12165) were used. Preliminary experiments comparing the addition of UAS-Dicer2 to enhance the RNAi potency of the UAS-RNAi lines showed no significant difference.

### Carbocyanine Dye Labeling

Single mechanosensory axons were labeled using lipophilic carbocyanine fluorescent dyes as previously described [16]. Two-day old female flies were anaesthetized and adhered to insect pins. Posterior scutellar (pSc) bristles were removed from the live flies, exposing the mechanosensory neuron in the bristle socket. The flies were decapitated and fixed in 3.7% paraformaldehyde in 0.2 M sodium bicarbonate buffer solution (pH 9.5) at 4°C. Following fixation, the fluorescent dyes DiI or DiD (Molecular Probes, Eugene OR) dissolved in ethanol at 20 mg/ml and 0.04 mg/ml, respectively, was injected into the bristle socket. Dye transfer was allowed to proceed for 36-48 hours, and the thoracic ganglion was then dissected out and imaged as a whole mount.

### Immunohistochemistry and Protein Quantification

We confirmed previous results of efficient knockdown of PlexinA using *GMR-Gal4*, *UAS-PlexA-dsRNA*, and by performing semi-quantitative fluorescence immunohistochemistry on *elav-Gal4*, *UAS-PlexA-dsRNA *animals using an anti-PlexinA antibody (kindly provided by Y. Rao lab) (Additional file 1, Figure S1) [11,19,40]. The entire central nervous system (CNS) from adult female *Drosophila *was dissected and fixed in 4% paraformaldehyde in phosphate buffered saline. The tissue was permeabilized in phosphate buffered saline with 1% TritonX-100 (PBT) and blocked with 1% bovine serum albumin. Samples were incubated in primary antibodies, rabbit anti-PlexinA and mouse anti-MAP1B (22c10, from Developmental Hybridoma Bank) at 1:1,000 dilution in 1% PBT at 4°C. Secondary antibodies used were Alexa Fluor^® ^488 goat anti-rabbit IgG and Alexa Fluor^® ^647 goat anti-mouse IgG (Life Technologies, Carlsbad, CA) at 1:300 dilution in 1% PBT. A Hoechst 33258 stain was performed prior to mounting the samples onto slides using ProLong Gold antifade mounting medium (Life Technologies, Carlsbad, CA). During imaging, exposure times were kept constant between samples, and experiments were performed in triplicate. For immunostaining of PlexinB protein, specific antibodies have thus far not been created.

Fluorescence intensity analysis was performed using custom-written software (available upon request) in MatLab (Mathworks, Natick, MA). Four regions of interest (ROIs) within each thoracic ganglion image were randomly chosen and the integrated pixel intensities for the channels measuring PlexinA levels and MAP1B levels were acquired. Pixel intensities were normalized to the MAP1B channel and averaged among the ROIs and are shown with standard deviations in Additional file 1, Figure S1. Images shown in the Figures have been adjusted only for brightness and contrast across the whole image, and adjusted equally among the different genotypes.

### Fluorescence Microscopy and Image Analysis

Fluorescent microscopy was conducted using a Zeiss AxioScopeA1 microscope under epi-fluorescence. Except for internal controls, only posterior scutellar axons were imaged. Images were acquired using a 40 × water objective, using red and far-red filters (for DiI and DiD, respectively) and a Zeiss AxioCam Mrm CCD camera with a 0.63 × adaptor so that the whole pSc axon (~135 μm × 245 μm) could be visualized. Images used for quantitative analyses were chosen on the basis of strong fluorescent signal within a single axon, low background signal and no damage to the ganglion from the dissection. A series of 15-20 images at different focal planes were taken to accurately represent the entire axonal arbor in the *z *dimension. Transmitted light images for each sample were taken to ensure that there were no occlusions on the surface of the thoracic ganglion and to normalize arbor sizes.

Quantitative image analysis was performed using custom-written software (available on request) in MatLab. All images were randomized and blinded before analysis. Images were analyzed as maximal intensity projections and adjusted for contrast and brightness prior to analysis. Lengths of all protrusions emanating from the axon after entry into the thoracic ganglion were measured and normalized to the width of the ganglion to account for differences in animal size, fixation artifacts, and imaging angle. Only branches greater than 4 μm in length were used to calculate total number of branches of a single pSc neuron, and to calculate total axonal branch length. A stereotypical skeleton of the wildtype pSc axonal arbor was designated by first identifying the axonal branches that were invariant among 21 *w*^- ^flies. Secondary and tertiary axonal branches that occurred at greater than 80% frequency were then chosen for the skeleton. These 16 branches ranged from 13 μm for the smallest branch average to 151 μm for the largest branch average.

Varicosity analysis was performed on a subset of the images acquired for highest quality. Contrast and brightness of the images were adjusted to threshold varicosities from the axon. Varicosities were counted as points along an axon that clearly extended beyond the width of the axon and had a higher fluorescence intensity. Axonal varicosities were used as a marker for putative synaptic contacts to obtain an estimate of synaptic number and location along the axon [26,27].

ANOVA analysis with Bonferroni correction was used to test for statistical significance (*p *< 0.05) in branch lengths between the different genotypes. Significant differences in branch numbers and varicosity numbers between groups were determined using the Mann-Whitney U-Test (*p *< 0.05), and the Kolmogorov-Smirnov two sample test was used to compare distribution functions. Statistical significance of frequency of axon guidance errors was determined using the χ^2 ^test (*p *< 0.05). Statistical significance in graphs were indicated by 1, 2, or 3 asterisks for *p *values less than 0.05, 0.01, and 0.001, respectively. Because PlexinA mutants produced opposing phenotypes from PlexinB mutants, all quantitative analyses between the two mutants were statistically significant and not indicated in the figures for clarity.

Averages for branch length and number and varicosity number were calculated along with variance and standard error. Results are reported as mean ± standard error of the mean (S.E.M.). For statistical analysis, *PlexA*^*f05547 *^and *PlexB*^*KG00878 *^mutants were compared to *w*^- ^wildtype controls, whereas all *455-Gal4*; *UAS-dsRNA *flies were compared to *455-Gal4 *alone as control. However, we did observe a significant difference between *w*^- ^and *455-Gal4 *flies in the number of branches (22.5 ± 0.3 and 24.4 ± 0.7, respectively) and the total lengths (906 ± 19 μm and 1045 ± 31 μm, respectively) (*p *< 0.05). The pSc skeleton of the *455-Gal4 *flies was also more variable than *w*^- ^flies and only 14 branches were consistently observed at > 80% frequency. Thus, the insertion of Gal4 into the 455 promoter region introduced a significant variable in axonal (but not varicosity) growth, but we do not believe this altered the interpretation of the data, especially for *455-Gal4*; *UAS-PlexB-dsRNA *flies (see **Results**).

Several lines of evidence demonstrate that our Plexin phenotypes in axonal branching and synapse development are not due to off-target effects of RNAi. First, analysis of *PlexA*^*LOF*^/+ heterozygous animals showed similar phenotypes to the RNAi knockdown, and these specific phenotypes were enhanced in combinations of the RNAi knockdown and heterozygotes. Second, using different RNAi lines targeting different sequences in the Plexins we found consistent and specific phenotypes. PlexinA and PlexinB are the only members of the *Drosophila *Plexins, and therefore the next closest gene target for the different dsRNA constructs used was the other Plexin (i.e., PlexinA dsRNA may have also targeted PlexinB mRNA due to sequence conservation). However, our RNAi experiments produced opposing axonal phenotypes, which makes it unlikely that the dsRNA non-specifically targeted other RNAs that was not the other Plexin to produce opposing phenotypes. Third, off target effects of RNAi would likely be enhanced with multiple copies of different RNAi constructs, but our experiments using *455-Gal4 *to drive both UAS-*PlexA*-*dsRNA *and UAS-*PlexB*-*dsRNA *in the same neuron revealed the opposite. Rather than having more severe pSc axonal phenotypes, neither of the opposing phenotypes were enhanced, and *455-Gal4*; *UAS-PlexA-dsRNA*/*UAS-PlexB-dsRNA *flies were indistinguishable from controls with respect to total arbor size, supporting our hypothesis for opposing roles of PlexinA and PlexinB.

## Competing interests

The authors declare that they have no competing interests.

## Authors' contributions

SQN and ADH performed the imaging experiments and analysis. ADH carried out the immunohistochemistry and performed the statistical analyses. SQN, ADH, and BEC participated in the design of the study and image analysis, and wrote the manuscript. All authors read and approved the final manuscript.

## Supplementary Material

Additional file 1 **Figure S1: PlexinA protein is widely expressed throughout the CNS and decreases after RNAi knockdown**. **A**, PlexinA is expressed along fibers within the thoracic ganglion. PlexinA protein distribution (green) was compared to MAP1B (red) in the thoracic ganglion in different genotypes. Negative control consisted of no PlexinA primary antibody (far left image), but with fluorescence secondary antibody and the MAP1B staining in the red channel. Scale bar, 50 μm. Inset, PlexinA protein was found along nerve tracts in punctuate staining. Scale bar, 10 μm. **B**, PlexinA levels are reduced using RNAi. To estimate PlexinA protein levels, semi-quantitative analysis was performed by measuring PlexinA pixel intensities normalized to MAP1B pixel intensities. Pan-neuronal knockdown of PlexinA using RNAi decreased PlexinA to near undetectable levels. *PlexA*^*LOF*^/+ heterozygous mutant did not have significantly different levels of PlexinA compared to wildtype, but our measurements are taken throughout the whole ganglion and do not reflect the PlexinA levels in single neurons. Click here for file

Additional file 2 **Figure S2: PlexinA suppresses varicosity formation cell-autonomously**. **A**, RNAi knockdown of PlexinA is specific for pSc neurons. Specificity of the *455-Gal4 *driver was verified by labeling a pSc neuron (green) expressing dsRNA against *PlexA *and a neighboring posterior Dorsocentral (pDc) neuron (red) within the same animal as an internal control. The pDc mechanosensory neuron forms its stereotypic wildtype branching pattern, whereas the pSc neuron expressing PlexinA RNAi displays excessive branches and varicosities (arrows). The pSc axon also failed to target the posterior primary axonal branch (asterisk). Scale bar, 50 μm. **B**, PlexinA reduction increases the number of axonal varicosities along single branches. PlexinA RNAi pSc neurons had 2.1 ± 0.1 (S.E.M.) varicosities per branch compared to 1.4 ± 0.1 in controls. The quantification of varicosities is performed only on large, high pixel-intensity unambiguous varicosities (i.e., a subset of those in the images) as an estimate of boutons. Scale bar, 15 μm. Click here for file
