# Supplementary material for: Opposing roles of PlexinA and PlexinB in axonal branch and varicosity formation
Source: Mol Brain. 2011 Apr 13;4:15. doi: 10.1186/1756-6606-4-15 (PMC3094289; doi:10.1186/1756-6606-4-15)
Supplement: Additional file 1 — Figure S1: PlexinA protein is widely expressed throughout the CNS and decreases after RNAi knockdown. A, PlexinA is expressed along fibers within the thoracic ganglion. PlexinA protein distribution (green) was compared to MAP1B (red) in the thoracic ganglion in different genotypes. Negative control consisted of no PlexinA primary antibody (far left image), but with fluorescence secondary antibody and the MAP1B staining in the red channel. Scale bar, 50 μm. Inset, PlexinA protein was found along nerve tracts in punctuate staining. Scale bar, 10 μm. B, PlexinA levels are reduced using RNAi. To estimate PlexinA protein levels, semi-quantitative analysis was performed by measuring PlexinA pixel intensities normalized to MAP1B pixel intensities. Pan-neuronal knockdown of PlexinA using RNAi decreased PlexinA to near undetectable levels. PlexALOF/+ heterozygous mutant did not have significantly different levels of PlexinA compared to wildtype, but our measurements are taken throughout the whole ganglion and do not reflect the PlexinA levels in single neurons. [file 1756-6606-4-15-S1.PDF]

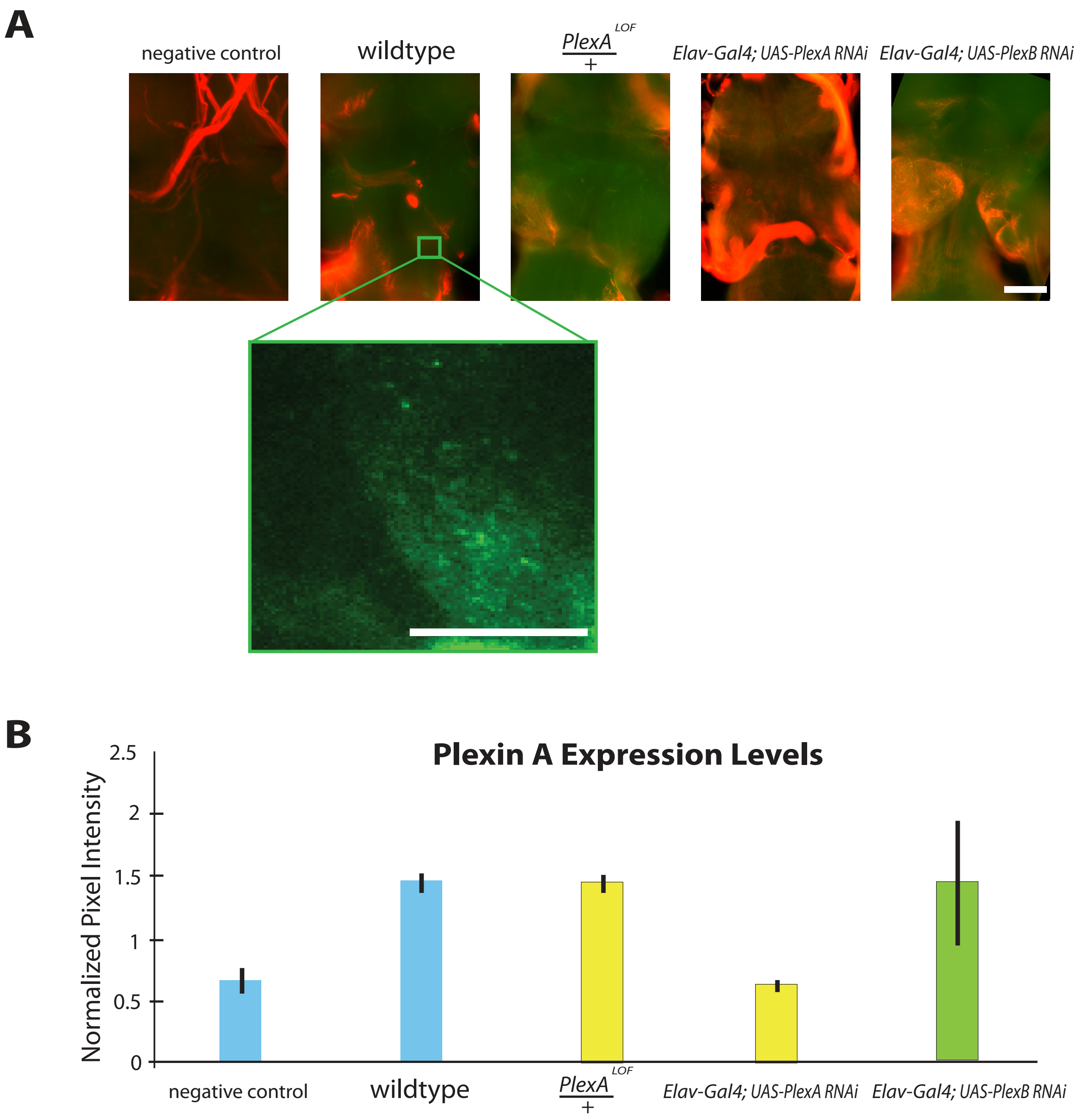

Additional file 1. Figure S1. PlexinA protein is widely expressed throughout the CNS and decreases after RNAi knockdown. A, PlexinA is expressed along fibers within the thoracic ganglion. PlexinA protein distribution (green) was compared to MAP1B (red) in the thoracic ganglion in different genotypes. Negative control consisted of no PlexinA primary antibody (far left image), but with fluorescence secondary antibody and the MAP1B staining in the red channel. Scale bar, 50 µm. Inset, PlexinA protein was found in punctuate staining along nerve tracts. Scale bar, 10 µm. **B, PlexinA levels are reduced using RNAi.** To estimate PlexinA protein levels, semi-quantitative analysis was performed by measuring PlexinA pixel intensities normalized to MAP1B pixel intensities. Pan-neuronal knockdown of PlexinA using RNAi decreased PlexinA to near undetectable levels. *PlexA*^{LOF}/+ heterozygous mutant did not have significantly different levels of PlexinA compared to wildtype, but our measurements are taken throughout the whole ganglion and do not reflect the PlexinA levels in single neurons.
