# Supplementary material for: Opposing roles of PlexinA and PlexinB in axonal branch and varicosity formation
Source: Mol Brain. 2011 Apr 13;4:15. doi: 10.1186/1756-6606-4-15 (PMC3094289; doi:10.1186/1756-6606-4-15)
Supplement: Additional file 2 — Figure S2: PlexinA suppresses varicosity formation cell-autonomously. A, RNAi knockdown of PlexinA is specific for pSc neurons. Specificity of the 455-Gal4 driver was verified by labeling a pSc neuron (green) expressing dsRNA against PlexA and a neighboring posterior Dorsocentral (pDc) neuron (red) within the same animal as an internal control. The pDc mechanosensory neuron forms its stereotypic wildtype branching pattern, whereas the pSc neuron expressing PlexinA RNAi displays excessive branches and varicosities (arrows). The pSc axon also failed to target the posterior primary axonal branch (asterisk). Scale bar, 50 μm. B, PlexinA reduction increases the number of axonal varicosities along single branches. PlexinA RNAi pSc neurons had 2.1 ± 0.1 (S.E.M.) varicosities per branch compared to 1.4 ± 0.1 in controls. The quantification of varicosities is performed only on large, high pixel-intensity unambiguous varicosities (i.e., a subset of those in the images) as an estimate of boutons. Scale bar, 15 μm. [file 1756-6606-4-15-S2.PDF]

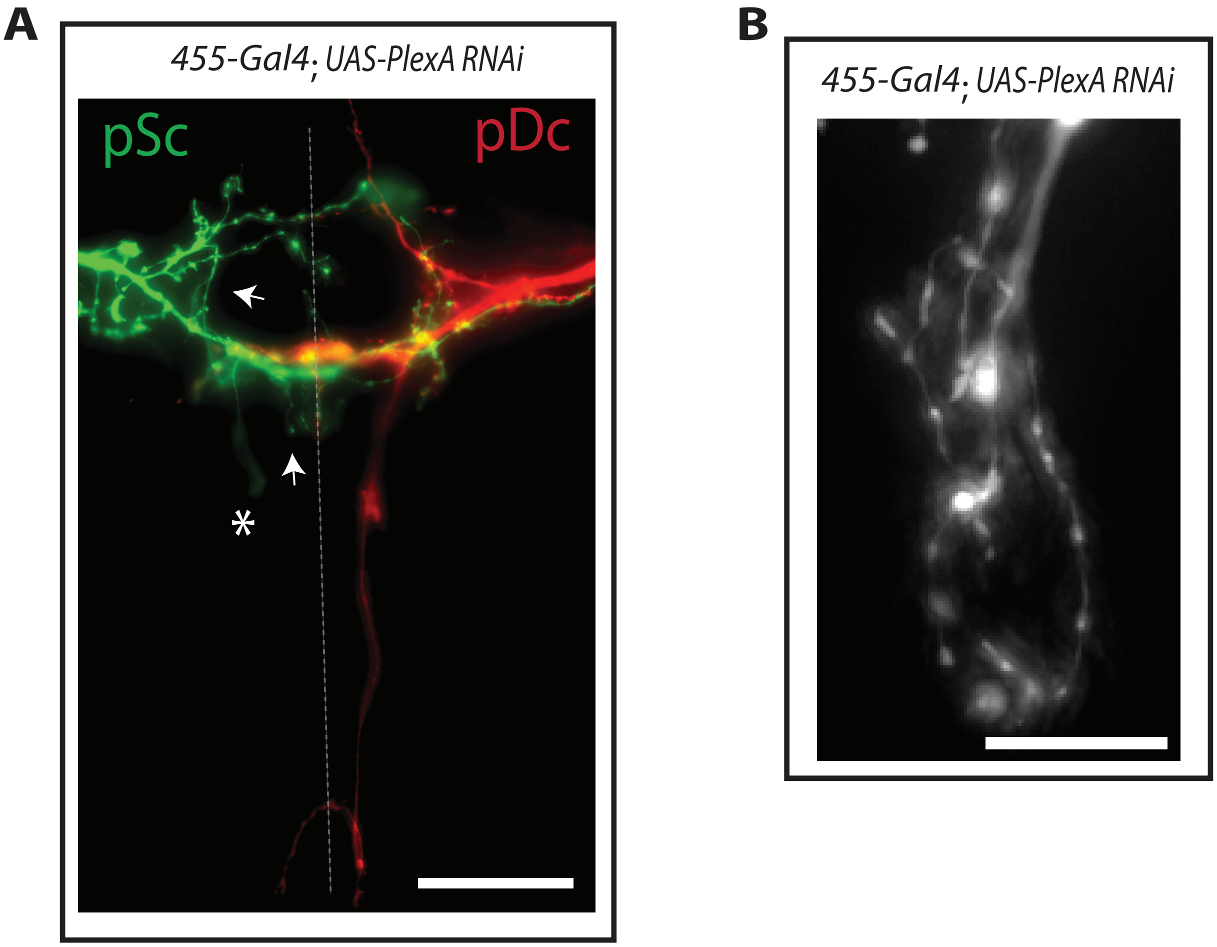

Additional file 2. Figure S2. PlexinA suppresses varicosity formation cell-autonomously. A, RNAi knockdown of PlexinA is specific for pSc neurons. Specificity of the *455-Gal4* driver was verified by labeling a pSc neuron (green) expressing dsRNA against *PlexA* and a neighboring posterior Dorsocentral (pDc) neuron (red) within the same animal as an internal control. The pDc mechanosensory neuron forms its stereotypic wildtype branching pattern, whereas the pSc neuron expressing PlexinA RNAi displays excessive branches and varicosities (arrows). The pSc axon also failed to target the posterior primary axonal branch (asterisk). Scale bar, 50µm. **B, PlexinA reduction increases the number of axonal varicosities along single branches.** PlexinA RNAi pSc neurons had 2.1 ± 0.1 (S.E.M.) varicosities per branch compared to 1.4 ± 0.1 in controls. The quantification of varicosities is performed only on large, high pixel-intensity unambiguous varicosities (i.e., a subset of those in the images) as an estimate of synaptic boutons. Scale bar, 15 µm.
